# Effect of a Compassion Cultivation Training Program for Caregivers of People With Mental Illness in Denmark: A Randomized Clinical Trial

**DOI:** 10.1001/jamanetworkopen.2021.1020

**Published:** 2021-03-08

**Authors:** Nanja Holland Hansen, Lise Juul, Karen-Johanne Pallesen, Lone Overby Fjorback

**Affiliations:** 1 Department of Clinical Medicine, Danish Center for Mindfulness, Aarhus University, Aarhus, Denmark

## Abstract

**Importance:**

Caregivers of people with mental illness are at increased risk of developing depression, anxiety, and stress.

**Objective:**

To investigate the effect of a compassion cultivation training (CCT) program on decreasing caregiver psychological distress.

**Design, Setting, and Participants:**

This waitlist-controlled randomized clinical trial was conducted in 2 different community settings in Denmark. Caregivers were excluded if they had a diagnosed and untreated mental illness, addiction, meditation practice, or current psychotherapeutic treatment. Enrollment occurred between May 2018 and March 2019. A repeated measurement model was used to examine the impact of the intervention. The primary analysis was based on the intention-to-treat principle. Data analysis was conducted from June 4 to July 7, 2020.

**Interventions:**

Participants were randomized 1-to-1 to an 8-week CCT course or waitlist control. Block randomization was used with 40 participants in each block.

**Main Outcomes and Measures:**

The main outcome was reduction in psychological distress, as measured by the Depression, Anxiety, Stress Scale (DASS). Baseline, postintervention, and 3- and 6-month follow-up measurements were collected.

**Results:**

Among 192 participants assessed for eligibility, 161 participants were included in the study (mean [SD] age, 52.6 [12.5] years; 142 [88.2%] women), with 79 participants randomized to the CCT intervention and 82 participants in the waitlist control group. At baseline, the mean (SD) DASS scores for the intervention vs control groups were 10.89 (8.66) vs 10.80 (8.38) for depression, 6.89 (6.48) vs 6.68 (5.33) for anxiety, and 14.96 (7.90) vs 15.77 (7.40) for stress. The CCT group experienced statistically significant improvement in the primary outcome in mean change from baseline vs the control group at postintervention (adjusted mean difference: depression, –4.16 [95% CI, –6.75 to –1.58]; *P* = .002; anxiety, –2.24 [95% CI, –3.99 to –0.48]; *P* = .01; stress, –4.20 [95% CI, –6.73 to –1.67]; *P* = .001), the 3-month follow-up (adjusted mean difference: depression, –3.78 [95% CI, –6.40 to –1.17]; *P* = .005; anxiety, –2.50 [95% CI, –4.27 to –0.73]; *P* = .006; stress, –3.76 [95% CI, –6.32 to –1.21]; *P* = .004), and the 6-month follow-up (adjusted mean difference: depression: –4.24 [95% CI, –6.97 to –1.52]; *P* = .002; anxiety, –2.12 [95% CI, –3.96 to –0.29]; *P* = .02; stress: –3.79 [95% CI, –6.44 to –1.13]; *P* = .005).

**Conclusions and Relevance:**

These findings suggest that CCT was superior to the waitlist control in supporting caregivers’ mental health. Statistically and clinically significant reductions in psychological distress were found and sustained at the 6-month follow-up. The improvements noted in this randomized clinical trial could serve to encourage implementation of future evidence-based programs for caregivers.

**Trial Registration:**

ClinicalTrials.gov Identifier: NCT03730155

## Key Points

**Question:**

Is a compassion cultivation training (CCT) intervention effective in decreasing psychological distress in informal caregivers of people with mental illness?

**Findings:**

In this randomized clinical trial including 161 caregivers randomized to a CCT program or waitlist group, caregivers who received CCT experienced significant improvements in depression, anxiety, and stress, and the improvements were maintained at 6-month follow-up.

**Meaning:**

Theses findings suggest that the CCT intervention was effective in decreasing psychological distress in caregivers of people with mental illness.

## Teaser

This randomized clinical trial examines the effect of a compassion cultivation training program on depression, anxiety, and stress among informal caregivers of people with mental illness in Denmark.

## Introduction

Suffering related to psychiatric disorders is present in patients and caregivers. Compassion can be understood as the willingness to feel suffering and to relieve suffering. Compassion is a practice of turning toward suffering rather than away,^1^ and it is a trainable skill.^2^ Training in compassion may be especially beneficial for caregivers, as enhancing compassion may promote mental health and reduce suffering. Caregivers are at an increased risk for mental health difficulties.^3,4,5,6,7^ Evidence-based programs to support caregivers’ mental health are needed.

Approximately 1 in 4 people provide care to a loved one.^3^ The economic contribution of informal care has been estimated as between 50% to 90% of the overall long-term cost of care in Europe,^3^ and as many as half of family caregivers may be at risk of developing depression.^3,7^

In Denmark, psychiatric disorders make up the largest disease burden. A total of 38% of the adult population categorize themselves as caregivers of someone with a mental illness, and 61% of caregivers experience psychological distress.^8^ It is estimated that the direct and indirect cost of poor mental health in Denmark is up to $9.17 billion.^9^

Compassion training has been found to be associated with decreased psychological distress and increased overall well-being.^10,11,12,13^ However, evidence-based programs for the effectiveness in the prevention of psychological distress for informal caregivers of people with mental illness are lacking. Two systematic reviews demonstrated that compassion training may promote mental health.^11,12^ However, the compassion interventions varied from 7 minutes to 8 weeks, with very few of the interventions being manualized, and not all included studies were randomized clinical trials (RCTs).^11,12^

A meta-analysis on compassion-based interventions with 21 RCTs,^13^ including either healthy adults or adults with a physical or mental illness, suggested a moderate effect size on measures of mindfulness, compassion, and self-compassion and decreased depression, anxiety, and psychological distress scores. Significant moderate effects were also found for well-being.^13^ However, there was great variability in the types of compassion-based interventions, their duration, and whether they were manualized or not.^13^ In a meta-analysis from 2017, Kirby et al^13^ concluded with suggestions for future research, including the use of larger samples sizes, an active control group, clinical samples, compassion-based questionnaires, and improved reporting and methods. This RCT sought to addresses some of these limitations by including a larger sample size, a high-risk population, 2 compassion-based self-report instruments (the 12-item Self-Compassion Scale-Short Form and Multidimensional Compassion Scale), and more rigorous methods by collecting repeated measurements at the 3- and 6-month follow-ups.

Compassion training programs may be thought of as preventive interventions that can decrease psychological distress and increase overall well-being. One such program is the manualized compassion cultivation training (CCT) program.^14^ Two RCTs^15,16,17,18,19^ including healthy adult participants have found significant increases in positive affect, cognitive reappraisal, acceptance, mindfulness skills, self-compassion, satisfaction with life, happiness, compassion, empathic concern, and identification with all humanity. Results of decreased negative affect, perceived distress, depression, suppression of emotion, negative rumination, and mind-wandering were also reported.^15,16,17,18,19^

Considering the potential for CCT to promote mental health and the lack of evidence-based programs for informal caregivers, our primary hypothesis was that the CCT program would decrease psychological distress in caregivers (eg, sibling, parent, spouse, adult child) of people with mental illness (any kind of mental disorder; eg, depression, anxiety, schizophrenia, bipolar disorder). The secondary hypothesis was that the CCT program would increase overall well-being in caregivers.

## Methods

### Study Design and Participants

This RCT included an intervention group and a waitlist control group. The study was conducted in 2 different community settings in Denmark, specifically Copenhagen and a more rural area in Jutland. Ethical approval was obtained at the Central Denmark Region Committee of Health Research Ethics (De Videnskabsetiske Komitéer for Region Midtjylland). All participants provided written informed consent. This study followed the Consolidated Standards of Reporting Trials (CONSORT) reporting guideline for clinical trials. The detailed trial protocol is available in Supplement 1.

Participants were all adults from Denmark. Participants were recruited through primary care physicians and social media and through national associations for caregivers, ads in local newspapers, and local 1-hour informational meetings on the topic of compassion. A total of 192 informal caregivers were assessed for eligibility through a telephone interview. Inclusion criteria included caregivers who were the parent, spouse, adult child, or sibling of a person with mental illness (all mental illnesses were included as described in *Diagnostic and Statistical Manual of Mental Disorders* [Fifth Edition] [*DSM-5*]^20^), aged 18 to 75 years, and Danish-speaking. The exclusion criteria included diagnosed and untreated mental illness, addiction, meditation practice, or current psychotherapeutic treatment. Of 192 assessed caregivers, 6 (3%) did not meet inclusion criteria, as they were either older than 75 years, they had an established meditation practice lasting more than 1 year, or they were receiving psychotherapy and did not feel they could manage without the help of a therapist.

 Participants signed a written informed consent form before answering the demographic and baseline questionnaire and before randomization.

### Randomization and Masking

Participants were randomized to CCT or a waitlist control (Figure) using a computer algorithm with predefined, concealed random numbers. The person creating the computer algorithm with predefined, concealed numbers was a university employee who administers the data software program, Redcap (Vanderbilt University), and was therefore blinded to study participants. We conducted block randomization with 40 participants in each block. The 40 participants in each block were all randomized at the same time, with 20 entering into the intervention group and 20 into the waitlist control group.

**Figure.**
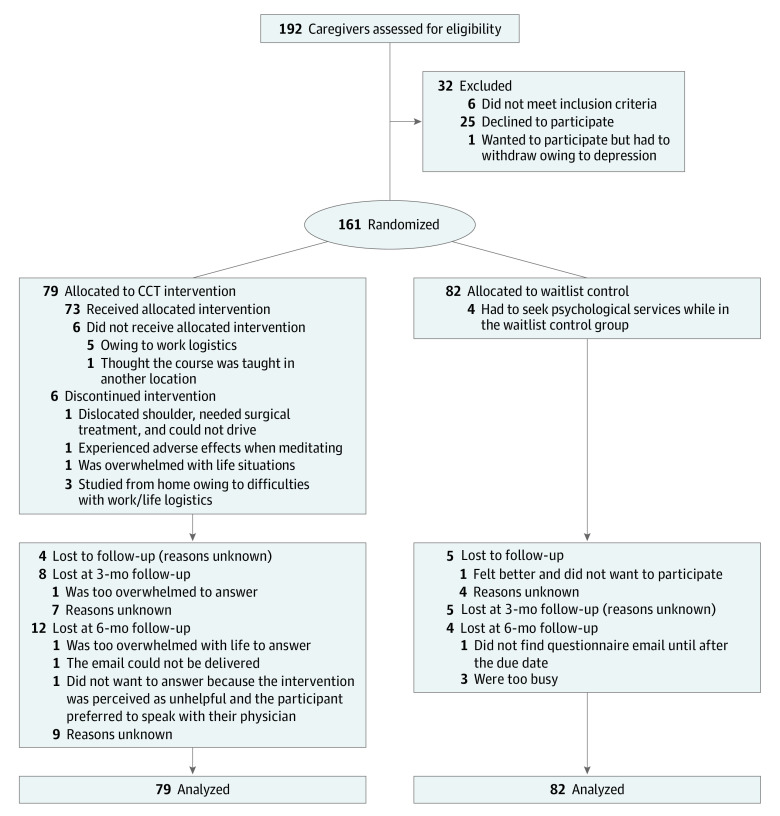
Flowchart of Participant Recruitment CCT indicates compassion cultivation training.

Participants enrolled in the trial received an email with a link to the questionnaires that they filled out online from their homes at postintervention and at the 3- and 6-month follow-ups. The data were collected in the RedCap secure university approved system. Once 6-month follow-up data were collected from the intervention and waitlist control groups, the waitlist control group received the CCT intervention.

Owing to the nature of the intervention, participants and the CCT instructor were aware of group allocation for the duration of the study. Data collection was remote, as participants answered the self-report questionnaires from their own homes via a link sent to them by email, and automatic, using the web-based Redcap software to ensure masking of outcome assessors. Everyone involved with the trial, except for the principle investigator and a research assistant, had no access to the data prior to analyzing the data.

### Intervention

The CCT program is a structured and manualized compassion training program that was developed at Stanford University Medical School in 2009.^14^ The program has a dual focus on training compassion and loving kindness for one’s own suffering and the suffering of others. The focus within CCT is to notice and pay attention to the suffering within oneself or others thereby becoming motivated to relieve that suffering. The program trains a variety of skills and techniques for emotional and mental well-being and is designed to promote qualities of compassion and empathy, and to cultivate kindness toward self, strangers, difficult people, and all sentient beings.^19^ The CCT program is a 8-week course (eTable 1 in Supplement 2). Participants meet for 2 hours each week, and the course incorporates practices of compassion, mindfulness, and meditation with scientific research on compassion and related topics in the fields of psychology and neuroscience along with contemplative thinking. Each week, participants engage in class discussions, formal meditations, and dyadic exercises. Participants are asked to meditate daily at home for 20 to 25 minutes using guided compassion meditations accessed through a website and engage in informal compassion practices, sent to the participants in an email each week. The CCT instructor adhered strictly to the CCT manual and received supervision on the teaching throughout the study by mindfulness and compassion practitioners with more than 20 years of experience.

### Outcomes

The primary outcome measure used was the 42-item Depression Anxiety Stress Scale (DASS).^21^ The DASS is scored by summing up the 14 items relevant for each subscale. Lower scores on each scale indicated less psychological distress.

There were 7 overall secondary outcome measures. We included the 10-item Perceived Stress Scale to measure how stressful a person perceives an event or situation in their life to be.^22^ Scores range from 0 to 40, with higher scores indicating higher perceived stress. The Brief Resilience Scale was used to assess the ability to bounce back or recover from stress (range, 6-10, with higher scores indicating more resilience).^23^ The World Health Organization 5-item Well-Being Index,^24^ measured the participant’s current mental well-being; scores range from 0 to 100, and higher scores indicate higher levels of well-being. A score of 50 or lower indicates a risk of developing stress or depression.^25^ The Emotion Regulation Questionnaire measured respondents’ tendency to regulate their emotions in 2 ways: either cognitive reappraisal or expressive suppression.^26^ The 12-item Self-Compassion Scale-Short Form was used to measure participants’ level of self-compassion.^27^ The Multidimensional Compassion Scale was used to measure 4 dimensions of compassion (ie, cognitive, affective, intentional, and motivational) and a total score (H. Jazaieri, P. R. Goldin, E. Simon-Thomas, D. Keltner, and R. Mendoza-Denton, unpublished data, May 2018). The 15-item Five Facet Mindfulness Scale measured 5 dimensions of mindfulness (ie, observing, describing, acting with awareness, nonjudging of inner experience, and nonreactivity to inner experience) and a total score.^28,29^ Higher scores indicate higher levels of mindfulness. All measures were Danish versions of the instruments, and the Brief Resilience Scale and Multidimensional Compassion Scale were translated into Danish using the World Health Organization guidelines for translating measurements.^30^

The CCT instructor assessed for safety and adverse events by informing participants that if any trauma resurfaced or other difficult emotions occurred during a session or at home when meditating, the participant should contact the CCT instructor (who is also a licensed psychologist). The participant and instructor would determine together the safest course of action.

### Statistical Analysis

The sample size was calculated using effect sizes from related publications,^11,15,16,17,18,19^ (respective η^2^ values, and Cohen *d*) with G*Power statistical software version 3.1 (Heinrich-Heine-Universität Düsseldorf). The power analysis gave an approximate value of a minimum of 77 participants in both groups when we expected a medium effect size of *d* = 0.5 (α = .05; power, 80%). A minimum of 77 participants per group allowed for an attrition rate of 20%, which gave us a minimum sample size of 64 participants each in the intervention and waiting list control groups.

Data were analyzed by a repeated measurement model with the systematic effect: age, sex, socioeconomic status, years as informal caretaker, schizophrenia (diagnosis of loved one), anxiety (diagnosis of loved one), time (4 time points), intervention, and interaction between time and intervention. We adjusted for schizophrenia, and anxiety as these 2 types of mental illness were overrepresented in the intervention or control group. The repeated measurement model is relatively robust to data missing at random, but we supplemented all analyses with sensitivity analyses representing 4 scenarios with data not missing at random. Missing outcomes were substituted with the model-based estimate, adding or subtracting 0.2 SD in the intervention or control group. We performed a loss to 6-month follow-up analysis for age, sex, educational level, income level, years of caretaking and baseline scores of DASS, Perceived Stress Scale, World Health Organization 5-item Well-Being Index, Brief Resilience Scale, and Emotion Regulation Questionnaire by *t* tests and χ^2^ tests (eTables 2-4 in Supplement 2). *P* values were 2-sided, and statistical significance was set at *P* = .05. Data were analyzed from June 4 to July 7, 2020. Full details of the statistical analysis plan are in Supplement 1.

## Results

Between May 18, 2018, to March 8, 2019, 192 caregivers were assessed for eligibility, and a total of 32 caregivers were excluded (6 for not meeting inclusion criteria, 25 declined to participate mainly owing to work/life balance, and 1 wanted to participate but became too depressed and withdrew). A total of 26 (14%) of the 187 caregivers eligible to participate in the study decided not to participate mainly due to logistical issues between work and home life. The final sample included 161 participants (mean [SD] age, 52.6 [12.5] years; 142 [88.2%] women), with 79 participants randomized to the CCT intervention and 82 participants in the waitlist control group (Figure). Demographic characteristics were similar for both groups at baseline (Table 1), and also after conducting an analysis of loss to at the 6-month follow-up (Table 1). There were no observed differences between groups on outcomes measures at baseline (Table 2). In the intervention group, 73 participants (92%) received 2 or more sessions of 8 total sessions, 65 participants (88%) completed 5 sessions, and 54 participants (74%) completed 6 or more sessions. One participant in the intervention group experienced adverse effects to meditation (prior trauma resurfaced). It was deemed safest to terminate participation, and a referral to see a psychologist was offered, which the participant declined.

**Table 1.** Demographic Characteristics of Caregivers at Baseline

Characteristic	No. (%)		
	Intervention group (n = 79)	Control group (n = 82)	Total (n = 161)
Sex			
Men	11 (14.1)	8 (9.7)	19 (11.8)
Women	68 (85.9)	74 (90.2)	142 (88.2)
Age, mean (SD), y	55.9 (13.3)	49.5 (10.8)	52.6 (12.5)
Educational level			
No high school	1 (1.3)	1 (1.2)	2 (1.2)
High school	4 (5.1)	2 (2.4)	6 (3.7)
Trade school	5 (6.3)	10 (12.2)	15 (9.2)
Short continuing education	8 (10.1)	3 (3.7)	11 (6.8)
Medium continuing education	43 (54.4)	25 (30.5)	68 (42.0)
Long continuing education	17 (21.5)	38 (46.3)	55 (34.0)
PhD	0	3 (3.7)	3 (1.9)
Other	1 (1.3)	0	1 (0.6)
Caretaking duration, y			
0-5	22 (28.2)	22 (27.1)	45 (28.1)
5-10	23 (29.5)	20 (24.7)	43 (26.9)
10-15	5 (6.5)	16 (19.8)	21 (13.1)
15-20	9 (11.5)	5 (6.2)	14 (8.8)
>20	19 (24.4)	18 (22.2)	37 (23.1)
Psychiatric disorder of person being cared for^a^			
Anxiety	18 (22.2)	35 (42.7)	53 (32.7)
ADHD	10 (12.7)	14 (17.1)	24 (14.8)
Autism	17 (21.5)	14 (17.1)	32 (19.8)
Bipolar disorder	9 (11.4)	12 (14.6)	21 (13.0)
OCD	6 (7.6)	12 (14.6)	18 (11.1)
Depression	19 (24.1)	21 (25.6)	40 (24.7)
Addiction	10 (12.7)	8 (9.8)	18 (11.1)
Personality disorder	8 (10.1)	13 (15.9)	21 (13.0)
PTSD	7 (8.7)	7 (8.5)	14 (8.6)
Schizophrenia	21 (26.6)	13 (15.9)	34 (21.0)
Eating disorder	7 (8.9)	3 (3.7)	10 (6.2)
Stress	6 (7.6)	9 (11.0)	16 (9.9)
Acquired brain injury	6 (7.6)	6 (7.3)	12 (7.4)
Other^b^	7 (8.7)	10 (12.2)	17 (10.5)

Abbreviations: ADHD, attention-deficit hyperactivity disorder; OCD, obsessive compulsive disorder; PTSD, posttraumatic stress disorder.

^a^ Caregivers often had loved ones with comorbid disorders; therefore, the percentages do not total 100.

^b^ Includes disruptive behavior, bodily distress syndrome, intellectual disability, psychogenic nonepileptic seizures, Parkinson disease, schizotypal disorder, attachment disorder, Tourette syndrome, dementia.

**Table 2.** Effect of CCT on the Primary Outcome of Depression, Stress, and Anxiety in Informal Caregivers of People With Mental Illness at Postintervention and 3- and 6-Month Follow-up

Measure	CCT Intervention			Control			Between-group difference, mean (95% CI)^a^	*P* value	Cohen *d*
	Caregivers, No.	Score, mean (SD)	Within-group change from baseline, mean (95% CI)^a^	Caregivers, No.	Score, mean (SD)	Within-group change from baseline, mean (95% CI)^a^			
Depression^b^									
Baseline	76	10.89 (8.66)	NA	79	10.80 (8.38)	NA	NA	NA	NA
Postintervention	68	7.84 (8.30)	−3.60 (−5.47 to −1.73)	76	11.28 (9.53)	0.56 (−1.22 to 2.34)	–4.16 (–6.75 to –1.58)	.002	0.66
3 mo	63	7.02 (8.14)	−3.68 (−5.60 to −1.76)	76	11.09 (10.93)	0.11 (−1.67 to 1.88)	–3.78 (–6.40 to –1.17)	.005	0.56
6 mo	54	6.61 (8.27)	−3.93 (−5.96 to −1.90)	72	11.40 (10.67)	0.31 (−1.50 to 2.12)	–4.24 (–6.97 to –1.52)	.002	0.45
Anxiety^b^									
Baseline	74	6.89 (6.48)	NA	82	6.68 (5.33)	NA	NA	NA	NA
Postintervention	67	5.10 (5.33)	−2.03 (−3.32 to −0.75)	73	6.73 (7.00)	0.21 (−0.99 to 1.40)	–2.24 (–3.99 to –0.48)	.01	0.51
3 mo	62	4.95 (5.51)	−2.01 (−3.33 to −0.69)	76	7.05 (6.95)	0.49 (−0.70 to 1.67)	–2.50 (–4.27 to –0.73)	.006	0.56
6 mo	53	5.55 (5.94)	−1.18 (−2.57 to 0.21)	72	7.43 (7.85)	0.94 (−0.26 to 2.15)	–2.12 (–3.96 to –0.29)	.02	0.30
Stress^b^									
Baseline	77	14.96 (7.90)	NA	78	15.77 (7.40)	NA	NA	NA	NA
Postintervention	65	10.65 (7.11)	−4.15 (−6.00 to −2.30)	77	15.81 (8.81)	0.05 (−1.67 to 1.77)	–4.20 (–6.73 to –1.67)	.001	0.74
3 mo	63	10.75 (8.35)	−3.90 (−5.78 to −2.03)	76	15.68 (10.36)	−0.13 (−1.87 to 1.60)	–3.76 (–6.32 to –1.21)	.004	0.56
6 mo	54	10.39 (7.74)	−4.17 (−6.15 to −2.19)	71	15.38 (10.03)	−0.38 (−2.15 to 1.39)	–3.79 (–6.44 to –1.13)	.005	0.43

Abbreviations: CCT, compassion cultivation training; NA, not applicable.

^a^ Adjusted for sex, age, educational level, years as informal caretaker, and diagnosis of schizophrenia or anxiety for patient.

^b^ Measured using the Depression, Anxiety, Stress Scale. Each subscale has a range of 0 to 14, with higher scores indicating more psychological distress.

At baseline, the mean (SD) DASS scores for the intervention vs control groups were 10.89 (8.66) vs 10.80 (8.38) for depression, 6.89 (6.48) vs 6.68 (5.33) for anxiety, and 14.96 (7.90) vs 15.77 (7.40) for stress. We found statistically significant CCT group effect on the primary outcome (DASS) for all 3 subscales (depression, anxiety, stress) compared with the control group at postintervention, (adjusted mean difference: depression, –4.16 [95% CI, –6.75 to –1.58]; *P* = .002; anxiety, –2.24 [95% CI, –3.99 to –0.48]; *P* = .01; stress, –4.20 [95% CI, –6.73 to –1.67]; *P* = .001), the 3-month follow-up (adjusted mean difference: depression, –3.78 [95% CI, –6.40 to –1.17]; *P* = .005; anxiety, –2.50 [95% CI, –4.27 to –0.73]; *P* = .006; stress, –3.76 [95% CI, –6.32 to –1.21]; *P* = .004), and the 6-month follow-up (adjusted mean difference: depression: –4.24 [95% CI, –6.97 to –1.52]; *P* = .002; anxiety, –2.12 [95% CI, –3.96 to –0.29]; *P* = .02; stress: –3.79 [95% CI, –6.44 to –1.13]; *P* = .005) (Table 2). The results of the secondary outcome measures showed statistically significant positive effects of the CCT intervention compared with the control group at all time points on overall well-being, resilience, self-compassion, mindfulness, cognitive reappraisal (emotion regulation), and statistically significant reduction on perceived stress and emotion suppression (emotion regulation). No effect was found on the awareness subscale of the Five Facet Mindfulness Questionnaire and on the Multidimensional Compassion Scale measuring compassion as a multidimensional construct (Table 3). Results of the primary and secondary outcomes remained statistically significant after conducting the sensitivity analysis (eTable 5 and eTable 6 in Supplement 2).

**Table 3.** Effect of CCT on Secondary Outcome Measures in Informal Caregivers of People With Mental Illness at Postintervention and 3- and 6-Month Follow-ups

Measure	CCT intervention			Control			Between-group difference, mean (95% CI)^a^	*P* value	Cohen *d*
	Caregivers, No.	Score, mean (SD)	Within-group change from baseline, mean (95% CI)^a^	Caregivers, No.	Score, mean (SD)	Within-group change from baseline, mean (95% CI)^a^			
BRS									
Baseline	77	3.11 (0.78)	NA	81	2.95 (0.81)	NA	NA	NA	NA
Postintervention	69	3.35 (0.85)	0.28 (0.14 to 0.43)	77	2.94 (0.80)	0 (−0.13 to 0.14)	0.28 (0.08 to 0.48)	.006	0.48
3 mo	63	3.46 (0.67)	0.35 (0.20 to 0.50)	77	2.97 (0.81)	0.04 (−0.10 to 0.17)	0.32 (0.12 to 0.52)	.002	0.60
6 mo	54	3.50 (0.89)	0.35 (0.20 to 0.51)	73	2.97 (0.88)	0.03 (−0.11 to 0.17)	0.32 (0.11 to 0.53)	.002	0.36
WHO-5									
Baseline	77	46.81(20.85)	NA	81	43.46 (19.10)	NA	NA	NA	NA
Post	68	55.76(19.74)	9.01 (4.51 to 13.52)	78	42.10 (19.41)	−1.17 (−5.40 to 3.07)	10.18 (3.99 to 16.36)	.001	0.74
3 mo	63	57.84(19.34)	10.51 (5.90 to 15.13)	77	46.39 (19.97)	3.12 (−1.14 to 7.37)	7.40 (1.12 to 13.67)	.02	0.53
6 mo	54	56.96(21.57)	8.91 (4.02 to 13.80)	73	44.82 (21.52)	1.38 (−2.95 to 5.71)	7.53 (1.00 to 14.05)	.02	0.35
PSS									
Baseline	79	21.20 (6.06)	NA	81	22.70 (6.60)	NA	NA	NA	NA
Postintervention	68	17.03 (6.57)	−4.48 (−5.87 to −3.09)	75	22.25 (7.41)	−0.27 (−1.60 to 1.06)	–4.21 (–6.14 to –2.29)	<.001	0.85
3 mo	63	17.10 (6.48)	−3.82 (−5.25 to −2.39)	76	21.62 (7.22)	−0.97 (−2.29 to 0.36)	–2.85 (–4.80 to –0.90)	.004	0.59
6 mo	54	16.85 (6.68)	−3.81 (−5.32 to −2.30)	73	20.85 (6.95)	−1.82 (−3.17 to −0.48)	–1.99 (–4.01 to 0.03)	.05	0.29
ERQ									
Reappraisal									
Baseline	79	25.57 (7.23)	NA	81	24.54 (6.71)	NA	NA	NA	NA
Postintervention	68	29.71 (6.17)	4.06 (2.68 to 5.45)	75	24.32 (7.20)	−0.07 (−1.40 to 1.26)	4.13 (2.21 to 6.05)	<.001	0.87
3 mo	63	29.68 (6.24)	3.48 (2.06 to 4.91)	77	25.39 (6.74)	0.84 (−0.48 to 2.15)	2.65 (0.71 to 4.59)	.007	0.58
6 mo	54	29.41 (6.84)	3.30 (1.79 to 4.80)	72	25.90 (6.98)	1.27 (−0.07 to 2.61)	2.02 (0.01 to 4.04)	.049	0.29
Suppression									
Baseline	79	12.68 (5.18)	NA	80	12.1 (4.34)	NA	NA	NA	NA
Postintervention	67	11.37 (3.80)	−1.14 (−2.16 to −0.12)	78	12.38 (5.53)	0.26 (−0.70 to 1.23)	–1.40 (–2.81 to 0)	.05	0.42
3 mo	63	11.32 (4.54)	−1.43 (−2.47 to −0.38)	77	12.05 (4.90)	0 (−0.97 to 0.96)	–1.42 (–2.85 to 0)	.05	0.43
6 mo	54	11.39 (4.82)	−1.28 (−2.38 to −0.17)	73	12.52 (4.61)	0.37 (−0.61 to 1.36)	–1.65 (–3.13 to –0.17)	.03	0.35
FFMQ									
Observing									
Baseline	77	10.23 (2.42)	NA	80	10.65 (2.70)	NA	NA	NA	NA
Postintervention	69	10.94 (2.45)	1.07 (0.58 to 1.57)	77	9.91 (2.69)	0.40 (−0.08 to 0.87)	0.68 (–0.01 to 1.36)	.05	0.37
3 mo	63	11.35 (2.49)	1.49 (0.97 to 2.00)	77	9.71 (2.72)	0.10 (−0.37 to 0.57)	1.39 (0.69 to 2.09)	<.001	0.75
6 mo	54	11.15 (2.37)	1.27 (0.72 to 1.81)	72	9.69 (2.78)	0.07 (−0.41 to 0.55)	1.20 (0.47 to 1.93)	.001	0.65
Describing									
Baseline	79	9.87 (2.75)	NA	81	9.63 (2.77)	NA	NA	NA	NA
Postintervention	68	11.25 (2.34)	1.17 (0.70 to 1.63)	78	10.56 (2.69)	−0.70 (−0.51 to 0.37)	1.24 (0.60 to 1.88)	<.001	0.69
3 mo	63	11.65 (2.03)	1.52 (1.05 to 2.00)	77	10.74 (2.59)	0.09 (−0.35 to 0.53)	1.43 (0.78 to 2.08)	<.001	0.87
6 mo	54	11.74 (2.51)	1.49 (0.99 to 2.00)	73	10.27 (2.70)	−0.19 (−0.64 to 0.26)	1.69 (1.01 to 2.36)	<.001	0.91
Awareness									
Baseline	79	9.42 (2.50)	NA	82	9.28 (2.60)	NA	NA	NA	NA
Postintervention	67	10.21 (2.35)	0.79 (0.23 to 1.34)	77	9.60 (2.56)	0.28 (−0.24 to 0.80)	0.50 (–0.25 to 1.26)	.19	0.29
3 mo	63	10.57 (2.19)	1.10 (0.54 to 1.66)	76	9.78 (2.83)	0.51 (−0.01 to 1.03)	0.59 (–0.18 to 1.36)	.13	0.33
6 mo	54	10.26 (2.51)	0.76 (0.17 to 1.36)	72	9.19 (2.68)	0.02 (−0.51 to 0.55)	0.74 (–0.06 to 1.54)	.07	0.40
Nonjudging									
Baseline	79	10.06 (2.45)	NA	80	10.05 (2.70)	NA	NA	NA	NA
Postintervention	68	11.43 (2.13)	1.49 (0.92 to 2.07)	75	10.17 (2.99)	0.47 (−0.06 to 1.01)	1.36 (0.59 to 2.14)	.001	0.74
3 mo	63	11.56 (2.39)	1.66 (1.05 to 2.27)	76	10.62 (2.73)	0.20 (−0.34 to 0.74)	1.02 (0.24 to 1.81)	.01	0.56
6 mo	54	11.69 (2.37)	1.36 (0.59 to 2.14)	73	10.26 (2.74)	1.37 (0.81 to 1.93)	1.46 (0.64 to 2.27)	<.001	0.81
Nonreacting									
Baseline	79	7.61 (2.10)	NA	80	7.2 (2.80)	NA	NA	NA	NA
Postintervention	68	9.35 (1.88)	1.75 (1.21 to 2.29)	78	7.18 (2.62)	0.08 (−0.43 to 0.60)	1.67 (0.92 to 2.41)	<.001	1.04
3 mo	62	9.23 (2.38)	1.57 (1.01 to 2.13)	77	7.65 (2.90)	0.51 (−0.01 to 1.02)	1.06 (0.30 to 1.83)	.006	0.56
6 mo	54	9.5 (2.77)	1.78 (1.19 to 2.37)	73	7.66 (2.68)	0.52 (0 to 1.05)	1.26 (0.47 to 2.05)	.002	0.65
Total									
Baseline	79	37.32 (5.96)	NA	77	37.31 (7.37)	NA	NA	NA	NA
Postintervention	65	42.26 (6.32)	5.01 (3.62 to 6.40)	74	37.45 (7.63)	0.18 (-1.15 to 1.50)	4.83 (2.91 to 6.75)	<.001	0.97
3 mo	62	42.90 (6.30)	5.70 (4.29 to 7.12)	76	38.80 (8.25)	1.50 (0.18 to 2.81)	4.21 (2.28 to 6.14)	<.001	0.81
6 mo	54	43.19 (7.32)	5.70 (4.22 to 7.19)	72	37.31 (7.75)	0.52 (−0.82 to 1.86)	5.18 (3.18 to 7.18)	<.001	0.69
SCS									
Baseline	76	36.45 (7.20)	NA	80	34.9 (8.13)	NA	NA	NA	NA
Postintervention	67	41.52 (7.57)	5.21 (3.66 to 6.76)	76	35.5 (8.71)	0.70 (−0.75 to 2.14)	4.52 (2.40 to 6.63)	<.001	0.78
3 mo	63	43.06 (7.90)	6.36 (4.78 to 7.94)	75	34.97 (8.85)	0.28 (−1.17 to 1.72)	6.08 (3.94 to 8.22)	<.001	1.03
6 mo	54	42.19 (8.56)	5.31 (3.64 to 6.99)	73	35.51 (8.37)	1.03 (−0.43 to 2.50)	4.28 (2.05 to 6.50)	<.001	0.51
MCS									
Cognitive									
Baseline	78	2.12 (0.77)	NA	81	2.04 (0.76)	NA	NA	NA	NA
Postintervention	68	1.82 (0.85)	−0.31 (−0.49 to −0.12)	77	2.03 (0.66)	0.01 (−0.16 to 0.19)	–0.32 (–0.58 to –0.63)	.02	0.59
3 mo	63	1.90 (0.85)	−0.20 (−0.40 to −0.01)	77	2.02 (0.72)	−0.01 (−0.19 to 0.16)	–0.19 (–0.45 to 0.07)	.15	0.34
6 mo	54	1.82 (0.69)	−0.29 (−0.49 to −0.09)	73	1.97 (0.59)	−0.02 (−0.20 to 0.16)	–0.27 (–0.54 to 0)	.05	0.59
Affective									
Baseline	78	2.43 (0.95)	NA	81	2.27 (0.95)	NA	NA	NA	NA
Postintervention	67	2.57 (0.93)	0.17 (−0.02 to 0.34)	76	2.39 (1.06)	0.18 (0.01 to 0.36)	–0.01 (–0.27 to 0.24)	.91	0.01
3 mo	63	2.42 (0.92)	0.03 (−0.16 to 0.22)	76	2.31 (1.06)	0.03 (−0.14 to 0.21)	0 (–0.26 to 0.26)	>.99	0
6 mo	54	2.50 (0.98)	0.04 (−0.16 to 0.24)	73	2.33 (0.96)	0.04 (−0.13 to 0.22)	0 (–0.27 to 0.27)	.99	0
Intentional									
Baseline	78	2.40 (0.83)	NA	80	2.45 (0.85)	NA	NA	NA	NA
Postintervention	68	2.01 (0.74)	−0.23 (−0.41 to -0.06)	77	2.25 (0.89)	0 (−0.17 to 0.17)	–0.23 (–0.48 to 0.01)	.06	0.40
3 mo	62	2.10 (0.66)	−0.12 (−0.30 to 0.07)	77	2.28 (0.82)	0.05 (−0.12 to 0.25)	–0.17 (–0.41 to 0.08)	.19	0.32
6 mo	53	2.13 (0.72)	−0.14 (−0.33 to 0.06)	72	2.29 (0.76)	0.04 (−0.13 to 0.21)	–0.18 (–0.44 to 0.08)	.17	0.34
Motivational									
Baseline	78	2.26 (0.78)	NA	81	2.22 (0.80)	NA	NA	NA	NA
Postintervention	67	2.56 (0.81)	0.15 (−0.04 to 0.33)	77	2.56 (0.91)	0.07 (−0.10 to 0.25)	0.07 (–0.18 to 0.33)	.57	0.11
3 mo	62	2.44 (0.67)	0.08 (−0.12 to 0.27)	77	2.59 (0.97)	0.10 (−0.08 to 0.28)	–0.02 (–0.29 to 0.24)	.86	0.03
6 mo	54	2.49 (0.82)	0.07 (−0.13 to 0.27)	73	2.57 (0.79)	0.07 (−0.11 to 0.24)	0 (–0.27 to 0.27)	.98	0
Total									
Baseline	75	2.22 (0.59)	NA	77	2.16 (0.62)	NA	NA	NA	NA
Postintervention	63	2.17 (0.63)	−0.01 (−0.14 to 0.12)	72	2.22 (0.65)	0.07 (−0.06 to 0.19)	–0.08 (–0.26 to 0.11)	.41	0.17
3 mo	60	2.15 (0.59)	0 (−0.14 to 0.13)	76	2.23 (0.62)	0.06 (−0.07 to 0.18)	–0.06 (–0.24 to –0.12)	.54	0.14
6 mo	53	2.18 (0.59)	0.03 (−0.17 to 0.11)	72	2.20 (0.48)	0.02 (−0.10 to 0.15)	–0.05 (–0.24 to 0.13)	.57	0.09

Abbreviations: BRS, Brief Resilience Scale; CCT, compassion cultivation training; ERQ, Emotion Regulation Questionnaire; FFMQ, Five Facet Mindfulness Questionnaire; MCS, Multidimensional Compassion Scale; NA, not applicable; PSS, Perceived Stress Scale; SCS, Self Compassion Scale; WHO-5, World Health Organization 5-item Well-Being Index.

^a^ Adjusted for sex, age, educational level, years as informal caretaker, and diagnosis of schizophrenia or anxiety for patient.

## Discussion

This randomized clinical trial found that the CCT intervention decreased symptoms of depression, anxiety, and stress in caregivers of people with mental illness. The effects remained at the 6-month follow-up. Positive effects of CCT compared with the control group were observed on overall well-being, resilience, self-compassion, and cognitive reappraisal, an emotion regulation strategy. Perceived stress was reduced as well as emotion suppression, another emotion regulation strategy. Of the 5 facets of mindfulness, 4 facets either increased, such as observing and describing, or decreased, such as nonjudging and nonreacting. No significant results were observed on the facet of awareness and on the 4 dimensions or total score of the Multidimensional Compassion Scale. This may be associated with the fact that these dimensions of compassion require more time to cultivate. Furthermore, there is a lack of consensus on how to define and measure the compassion for others construct.^13^

Our findings that CCT decreased psychological distress and increased overall well-being are in line with previous RCTs on CCT.^15,16,17,18,19^ Comparing the results of our study with previous caregiver intervention studies, the literature does not present a clear picture of what kind of intervention is most helpful for caregivers of people with mental illness. An 8-week manualized group intervention with psychoeducation,^31,32^ a caregiver education and social support program,^33^ a cognitive behavioral therapy group,^34,35^ a mindfulness-based stress reduction program,^36,37^ and a yoga and compassion meditation program^38^ have shown improved mental health at the end of treatment, but have either not tested the effect at the 6-month follow-up or not found significant results. An individual therapy session intervention^39^ with 6 months of follow-up has produced similar results as our CCT study.

Systematic reviews and meta-analyses of caregiver interventions and mental health have found different results based on the type of mental illness. Psychoeducational and group interventions may be most helpful to people with severe mental illness,^40^ while individual in-home multicomponent or psychoeducational interventions may be most helpful to caregivers of people with dementia.^41^ Therefore, there is a great need for structured and systematic trials that replicate trials like this study of a CCT program with an active control group to understand whether the results observed here continue to be superior when an active control group is included, and investigate what mechanisms are the most helpful in interventions for informal caregivers.

### Strengths and Limitations

This study’s strengths include a well-powered, rigorously conducted RCT with 6-month follow-up data, a high-risk population that included citizens with broad inclusion criteria from 2 different geographically settings in Denmark, which enhances the generalizability of the results to a broad target population. However, we did not perform subgroup or effect modification analysis to investigate whether the effect differed given some characteristics (eg, diagnosis of loved one). It was outside the scope of this RCT, and the RCT was not powered to do so. The CCT intervention is a manualized program, the psychologist was experienced in delivering the CCT program and was supervised.

This study also has some limitations, including differential loss to follow-up at 6 months, which could bias the results. To mitigate against this possibility, we conducted sensitivity analysis for the primary and secondary outcomes and observed that all results remained statistically significant. A waitlist design has the potential to overestimate intervention effects owing to issues of expectancy. Research has shown larger effects sizes in favor of this type of control group when compared with treatment as usual.^35^ As the trial did not include an active control group, we are not able to determine whether the CCT program had a greater effect compared with another type of intervention. However, a waitlist comparator may be more reflective of what usually happens in the absence of an intervention like CCT. We cannot rule out the potential bias caused by the nonblinding of the intervention allocation, as expectations may have been influenced by their allocation. Another limitation was that 2 teams of caregivers (eg, 2 parents sharing caregiving for an adult child with mental illness) were included in the trial. There is a possibility that a cluster effect may exist. The parental team was randomized into the control group and the mother/daughter team was randomized into the intervention group. We used self-reported outcome measures, and cannot exclude the possibility that information bias is present, although all participants in the waitlist group, regardless of whether they answered the self-report questionnaires, were invited to participate in the CCT group after collecting 6-month follow-up data.

## Conclusions

This RCT found that the CCT intervention improved the mental health of a mixed group of caregivers whose loved ones had a variety of mental illnesses, with lasting effect of 6 months. The results suggest that compassion is a trainable skill that promotes mental health and that CCT could be taught in a group format reducing societal cost. According to WHO, depression is the leading cause of disability worldwide, and perceived stress is an independent risk factor for increased illness and mortality.^42^ Policy makers and health care professionals have few options in offering caregiver’s evidence-based interventions that help improve their mental health.^43^ Future research should replicate findings and compare the intervention with an active control. Furthermore, a potential mediating effect of the CCT intervention on compassion for self and mindfulness on psychological distress should be explored and future research should investigate whether there is a difference in the effect depending on caregiver status, type of mental disorder, and length of intervention.

## Notes

Supplement 1. **Trial Protocol and Statistical Analysis Plan**

Supplement 2. **eTable 1.** Steps of the Compassion Cultivation Training Program **eTable 2.** Loss to 6-Month Follow-up Analysis of Included and Missing Participants in Both the CCT Intervention and Waitlist Control Groups at 6-Month Follow-up **eTable 3.** Loss to 6-Month Follow-up Analysis of Included and Missing Participants in the CCT Intervention Group at 6-Month Follow-up **eTable 4.** Loss to 6-Month Follow-up Analysis of Included and Missing Participants in Waitlist Control Group at 6-Month Follow-up **eTable 5.** Sensitivity Analysis of Primary Outcome of Psychological Distress **eTable 6.** Sensitivity Analysis of Secondary Outcome of Overall Well-Being

Supplement 3. **Data Sharing Statement**
