# Supplementary material for: Effect of a Compassion Cultivation Training Program for Caregivers of People With Mental Illness in Denmark: A Randomized Clinical Trial
Source: JAMA Netw Open. 2021 Mar 8;4(3):e211020. doi: 10.1001/jamanetworkopen.2021.1020 (PMC7941195; doi:10.1001/jamanetworkopen.2021.1020)
Supplement: Supplement 1. — Trial Protocol and Statistical Analysis Plan [file jamanetwopen-e211020-s001.pdf]

Project title: Compassion Cultivation Training (CCT): A preventive Intervention for caregivers of people who suffer from a mental illness

Project Group:

Nanja Holland Hansen, licensed psychologist and Ph.D. student, Danish Center for Mindfulness, Department of Clinical Medicine, Aarhus University

Lise Juul, Ph.D. Associate Professor at Danish Center for Mindfulness, Department of Clinical Medicine, Aarhus University

Karen-Johanne Pallesen, Ph.D. Associate Professor at Danish Center for Mindfulness, Department of Clinical Medicine, Aarhus University

Lone Overby Fjorback, Ph.D., MD. Associate Professor at Danish Center for Mindfulness, Department of Clinical Medicine, Aarhus University

Project title: Compassion Cultivation Training (CCT): A preventive intervention for caregivers of people who suffer from a mental illness

Background:

The caregivers of people who suffer from mental illness are at raised risk for mental health difficulties (Sorrell, 2014, Stansfeld, 2014, Clark et al., 2011) such as depression, stress and anxiety. Physical health can also be problematic (Clark et al., 2011). In Denmark alone, there are approximately 1.6 million who categorize themselves as caregivers of someone with a mental illness. Of those caregivers, a small, but significant, proportion provides care to a person with a mental illness for 15 hours per week or more (Bedre Psykiatri (Better Psychiatry).

Informal caregiving has come about, as a result of people living longer, and the deinstitutionalization of the health care system. The formal care that once was provided by nurses and other healthcare personnel has now, to a large degree, been given to family members. Caregivers become “hidden patients” who are struggling with their own psychological and physical health as well as providing care for someone with mental illness (The Lancet Editorial, 2017).

A study demonstrated that 25-50% of caregivers develop depression (Clark et al., 2011). If these figures can be translated to the Danish context, 40.000 – 80.000 Danes can be expected to develop depression as a direct result of caring for a loved one with mental illness. A 2012 analysis of National Statistics from Eurostat demonstrates, that the direct and indirect costs per person with depression in Europe is €3034 (Olesen, et al., 2012). Consequently, it is estimated that the cost to Danish society related to the increased incidence of depression among caregivers of the mentally ill amounts to between 120 – 240 million Euros. Det Nationale Forskningscenter for Arbejdsmiljø (The National Research Center for Work Milieu) estimate that the total direct and indirect costs due to mental health related issues in Denmark are 55 billion Danish kroner annually (Danish Mental Health Fund, 2017). Together, these findings provide impetus to study interventions to support caregivers, particularly preventive interventions that increase their psychological and physical health, thereby decreasing the economic burden on society (Jacobsen, 2011).

There is a call for research-based interventions for caregivers (Northouse et al., 2010). Systematic and meta-analysis reviews of intervention programs for caregivers have found conflicting evidence for the effectiveness of interventions programs (Knight et al., 1993) stating that there were too many interventions and too little information on what mechanisms within the interventions were helpful. Others suggest that interventions that specifically address the needs of informal caregivers lead to improvements in their quality of life, a decrease in burden and psychological distress (Yesufu-Udechuku et al., 2015, Northouse et al., 2010). Sörensen et al., (2002), concluded "interventions are on average, successful in alleviating burden and depression, increasing general subjective well-being, and increasing caregiving ability/knowledge. To our knowledge, no preventive interventions aiming at providing caregivers with the skills necessary to increase their emotional resiliency to the caregiver burden have been applied in Denmark.

Compassion training research:

There is a strong and growing interest in the scientific community to explore how compassion is trained, defined, measured and implemented into various settings (Kirby, 2016). Clinical scientists are examining the impact compassion training has on emotional experience, emotion regulation, and psychological flexibility" (Goldin & Jazaieri, 2017). Thus far, research on compassion training point to the potential of being a tool to enhance and sustain mental and physical health (Goldin & Jazaieri, 2017, Kirby, 2016, Hoffman et al., 2011). A systemic review on the impact of compassion training on the treatment of psychopathology the authors concluded that compassion interventions may be effective in treating a broad array of mental health issues such as improvement in psychological distress, levels of positive and negative affect, the frequency and intensity of positive thoughts and emotions, empathic accuracy, and interpersonal skills (Shonin et al., 2015). Another review found that compassion training was associated with reduction in stress and subjective distress, increased immune response, and improvements in the activation of brain areas that are involved in processing emotions and empathy (Hoffman et al., 2011).

Studies on compassion training further found that feeling compassionate decreased heart rate and feeling distressed increased heart rate (Gu et al., 2017), and that displaying compassionate concern for

others lowered cortisol reactivity and blood pressure, increased heart rate variability, allowed participants to receive greater support from others, and increased participant's positive affect (Cosley et al., 2010). Other studies have found that a brief compassion exercise increased feelings of social connection and positivity towards strangers (Hutherson et al., 2008).

Klimicki et al., (2014) found that the participants receiving empathy training showed brain activation in areas associated with pain and empathy and when the same participants afterwards received compassion training the effects reversed not only by strengthening their positive affect but also by activating brain areas associated with affiliation and love. This research suggests that being exposed to the suffering of others may lead to two different and distinct emotional reactions: 1) *empathetic distress*, which when being exposed to empathetic distress over a long period of time, (such as loved ones suffering from a mental illness) negative feelings and withdrawal emerge and give rise to negative health outcomes, and 2) *compassionate response* that is based on other-oriented and positive feelings and activates pro-social motivational behavior. In sum, this body of research suggests that compassion training may benefit our mental and physical health by improving emotion regulation skills, interpersonal and social relationships, and by activating our parasympathetic system which aims to soothe and calm thereby promoting better physical health.

Compassion Cultivation Training (CCT):

Compassion is often defined as the feeling that arises when we witness someone suffering and we feel motivated to help the person who is suffering (Goetz et al., 2010, Kirby, 2016). Compassion Cultivation Training (CCT) is a comprehensive compassion training program, with a dialectical focus on training compassion for one's own suffering and the suffering of others (including a loved one, a stranger, a difficult person, and all living beings). While the foundation of compassion training is rooted in mindfulness (the paying attention to the present moment without judgment), the focus within compassion training is to notice and pay attention to the suffering within oneself or others thereby becoming motivated to relieve that suffering. The CCT program trains a variety of skills and techniques for emotional and mental well-being and is designed to promote qualities of compassion and empathy, and to cultivate kindness towards self, others and difficult people (Goldin & Jazaieri, 2017).

The theoretical model used in this study is the Process Model of Emotion Regulation developed by Gross & John (2003). Underlying this model is a conception of an emotion-generative process. This conception considers that emotions start with an evaluation of emotion cues. When the emotion cue is attended to and evaluated in different ways, the emotion cues then set in motion a coordinated set of responses that include experiential, physiological and behavioral systems. When the response tendencies arise, they may be regulated in different ways (Gross & John, 2003).

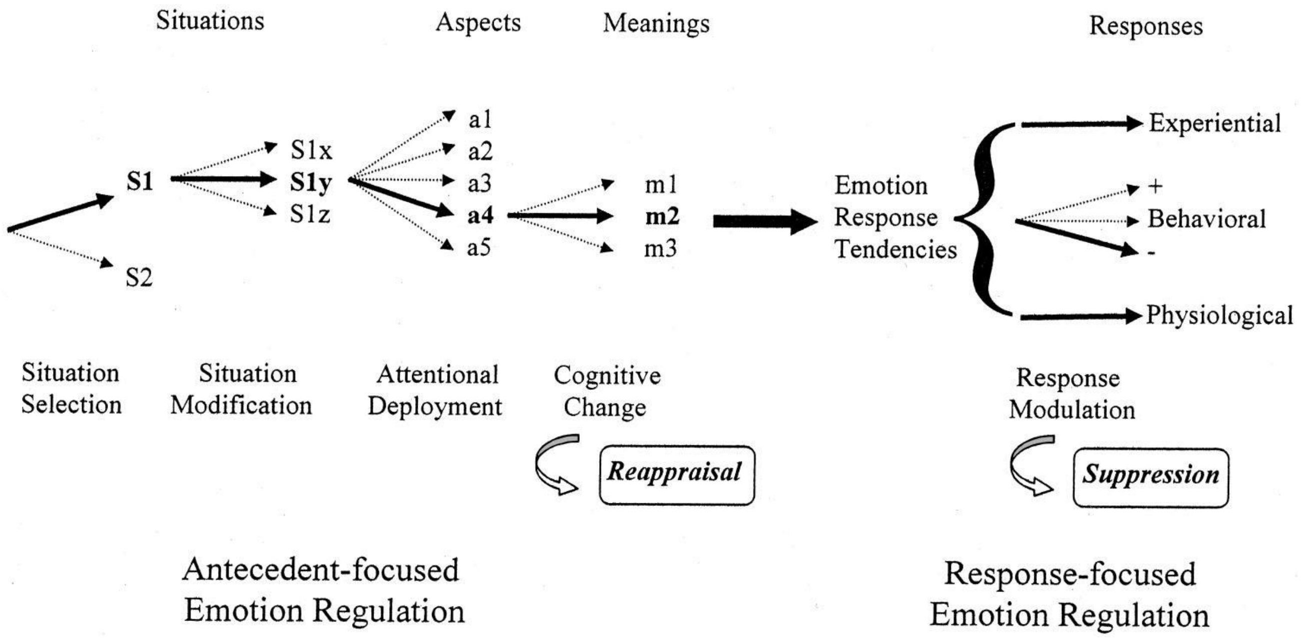

Reprinted from “Emotion Regulation in Adulthood: Timing Is Everything,” by J. J. Gross, 2001, *Current Directions in Psychological Sciences, 10,* p. 215. Copyright 2001 by Blackwell Publishers. Reprinted with permission.

According to the model, emotions can be regulated at five points: 1) selection of the situation, 2) modification of the situation, 3) deployment of attention, 4) change of cognitions, and 5) modulation of the experiential, physiological, and behavioral responses (Gross & John, 2003).

The first four responses are *antecedent- focused:* Things we do before an emotion response tendency has become fully activated and has changed our behavior and physiological response. The fifth response is *response-focused:* Things we do once an emotion is already in process and after the

response tendencies have already been created (Gross & John, 2003). One such response- focused strategy is emotion suppression and can be used as an emotion regulation strategy when faced with difficult emotions.

In a study by Gross & John (2003), emotion suppression in participants produced feelings of inauthenticity, masking of inner feelings, confusion about what they were feeling, less successful at mood repair, viewed their emotions less favorably and accepting, experienced more negative emotions and less positive emotions, more reluctant to share how they felt with others and avoided close relationships (Pendry & Wright, 2016). The application of this theory to compassion training is that in training caregivers' ability to regulate and accept difficult thoughts and emotions will allow for less emotion suppression and greater acceptance of their own emotional responses, leading to a decrease in overall psychological distress.

Current research on CCT:

Preliminary research findings on CCT have yielded several results: 1) increases in compassion for others and decreases in fear of compassion for self and others, 2) home meditation practice predicted CCT related changes such as a decrease in worry, 3) significant changes in emotion experience such as increased positive affect, decreased negative affect and perceived distress, 4) significant changes in emotion regulation such as increased cognitive reappraisal and acceptance and decreased suppression of emotion, 5) significant changes in cognitive regulations such as increased mindfulness skills, decreased negative rumination and mind-wandering (Jazaieri et al., 2013, 2014, 2015).

Results further indicate that when 1) participants have practiced a compassion meditation that same day, the probability of the participant having an other-focused caring behavior increase by 3.5 times, 2) having practiced a compassion meditation that same day increase the probability of self-caring behavior by 6.5 times, and 3) having engaged in self-care behavior that day participants are 9.3 times as likely to engage in an other-care behavior (Goldin & Jazaieri, 2017). Research on women with chronic pain receiving the CCT course found reduced pain severity and anger, and increased acceptance of pain (Chapin et al., 2014).

Two 2017 studies on the effects of CCT demonstrated significant improvements on self-compassion, mindfulness, and interpersonal conflict (Scarlet et al.,), increased skills in regulating affective experiences while simultaneously shifting towards not influencing affective states, indicating that participants of the CCT program may be more willing or able to accept their difficult emotions instead of suppressing or avoiding them allowing them to become self-efficacious in promoting acceptance of their affective experience (Jazaieri et al., 2017). Thus far, CCT has been studied in the general population, in people with chronic pain, and health care professionals (Jazaieri, 2013, 2014, 2015, 2017, Chapin et al., 2014, & Scarlet et al., 2017). The research on the CCT program is promising, yet more rigorous trials are needed to assess the effectiveness and utility of compassion interventions (Kirby, 2016).

The literature on interventions for caregivers along with compassion training suggests that the different components of psycho-educational group processes, training in compassion and mindfulness alongside meditation and dyadic exercises, may be helpful components in decreasing psychological distress, increase well-being and social connectedness (Hutcherson et al., 2008, Kok & Singer, 2016). The CCT program may be a helpful preventive intervention for caregivers of the mentally ill. Therefore, this study is of high value as it aims to bring skills to a "hidden" group within the Danish population of informal caregivers so that they may be better equipped to take care of their own emotional health. The skills they will acquire through the CCT course may allow caregivers greater flexibility in regulating emotions and greater acceptance of difficult emotions, increasing their emotional resiliency and decreasing psychological distress. This will not only benefit themselves and their loved one suffering from a mental illness but it will also benefit Denmark economically.

Aim:

This study will begin a novel line of research on CCT in Denmark as a preventive intervention for caregivers of people suffering from a mental illness. The primary aim of the study is to investigate the effectiveness of a Compassion Cultivation Training (CCT) course.

Hypothesis 1: It is hypothesized that caregivers in CCT will reduce psychological distress, relative to control participants, as measured by the Depression Anxiety Stress Scale (DASS: Lovibond &

Lovibond, 1995) at baseline (T0), post intervention (T1), 3-month (T2) and 6-month (T3).

Hypothesis 2: It is hypothesized that caregivers in CCT, relative to control participants, will increase overall well-being, compassion for self and others, resilience, mindfulness, and show greater acceptance of difficult emotions and decrease emotion suppression perceived stress, as measured by World health Organization Well-Being Index (WHO-5: Beck, 2012), Self-Compassion Scale Short Form (SCS-12: Raes et al., 2011), Multidimensional Compassions Scale (MCS: Jazaieri et al., 2018), Perceived Stress Scale, (PSS: Cohen et al., 1983), The Emotion Regulation Questionnaire (ERQ): Gross & John, 2003), Five Facet Mindfulness Questionnaire (FFMQ-15: Baer et al., 2006) measured at T0, T1, T2, and T3.

Hypothesis of Mechanisms: Improvements on these skills will mediate the effects of treatment and outcome. Specifically: a) increase in compassion for self and others (SCS-12 and MCS) will mediate the effects of emotion regulation skills (i.e. greater acceptance of difficult emotions and therefore less suppression of difficult emotions) and b) increase in emotion regulation skills (ERQ) (i.e. greater acceptance of difficult emotions and therefore less suppression of difficult emotions) will mediate the effects of psychological distress (DASS) in informal caregivers.

Methods

Research design

The effect of CCT will be evaluated in a parallel randomised controlled trial including 77 participants in the intervention group and 77 in a wait-list group.

Participants and Recruitment: Participants will be recruited through primary care physicians, the national association for caregivers; Bedre Psykiatri, Landsforeningen for pårørende (Better Psychiatry, national association for caregivers), through Psykiatrifonden (The Danish Mental Health Fund) and through social media such as Facebook, Twitter, and the Danish Center for Mindfulness website. In addition, recruitment will be carried out through CSV’s website, local newspapers and their collaborators. Participants who meet the inclusion and exclusion criteria will be recruited.

Eligibility Criteria

***Inclusion criteria**: a)* caregiver male and female (parent /spouse/sibling/adult child) of a person with a mental illness, *b)* 18 - 75 years of age, and *c)* Danish speaking.

***Exclusion criteria**: a)* diagnosed and untreated mental illness, *b)* addictions, *c)* meditation practice (studies have shown that people who are long-term meditation practitioners are more resilient and have greater psychological well-being (Lykins & Baer, 2009). Therefore, people with 1 year or more of prior formal meditation practice, will not be eligible for the study as we cannot rule out whether their scores are due to their long-term practice or to the CCT intervention), *d)* or current psychotherapeutic treatment.

Randomization: After informed consent and T (0) measures, participants will be randomized to either CCT (N=77) or WLC group (N=77) using a computer algorithm with predefined, concealed random numbers. An independent statistician will manage the randomization.

Procedure: Eligible participants, meeting all study criteria, are asked to participate in the RCT. All participants will be given psychological, and demographic measurements at baseline (T0), and psychological measures at post intervention (T1), 3-month follow-up (T2) and 6-month follow-up (T3), The study will be registered in ClinicalTrails.gov before commencement. The investigators will specifically ask that the participants in the WLC group do not start any other intervention during the study period.

Power: The sample size was calculated using effect sizes from related publications (Kirby et al., 2017; Jazaieri et al., 2015, 2013, 2012, Kuhlmann et al., 2015, Galante, et al., 2014, & Brito-Pons, 2014), respective η-square-values, and Cohen's d) with G*Power. The power analysis gave an approximate value of a minimum of 77 participants in both groups where we expect a medium effect size of .5 Cohen's d (alpha .05, power 80%). A minimum of 77 participants per group allows for an attrition rate of 20%, which will give us a minimum sample size of 64 participants per group. Four groups of approximately 20 participants per group will be given the CCT intervention.

Measures

Primary psychological measures:

Depression Anxiety Stress Scales (DASS: Lovibond & Lovibond, 1995). The DASS is a 42-item self report instrument designed to measure the three related negative emotional states of depression, anxiety and tension/stress.

Secondary psychological measures:

The World Health Organization Five Well-Being Index (WHO-5: Bech, 2012). The WHO-5 index is a short self-reported measure of current mental wellbeing that consists of five statements, which respondent's rate according to the 6-point Likert scale.

Brief Resilience Scale (BRS: Smith et al., 2008). The BRS is a 6-item scale assessing the ability to bounce back or recover from stress.

Perceived Stress Scale (PSS: Cohen et al., 1983). The PSS assesses the perceived stress within the last month. It is a 10 item self-report questionnaire using a 5 point-Likert scale

The Emotion Regulation Questionnaire (ERQ: Gross & John, 2003). The ERQ is a 10-item scale designed to measure respondents' tendency to regulate their emotions in two ways: 1. Cognitive Reappraisal and 2. Expressive Suppression. Respondents answer each item on a 7-point Likert-type scale.

Self-compassion Scale-12 (SCS-12: Raes et al., 2011). The SCS is a 12-item scale is designed to measure respondent's level of self-compassion. Respondents answer on a 5-point Likert scale.

Multidimensional Compassion Scale (MCS, Jazaieri et al., in prep). The MCS scale is a general measure of compassion with four components: Cognitive, affective, intentional, and motivational. The scale is comprised of 16 questions and respondents answer on a 7-point Likert scale.

Five Facet Mindfulness Scale-15 (FFMQ-15: Baer et al., 2006). The FFMQ is a 15-item scale measuring mindfulness. Respondents answer on a 5-point Likert Scale.

Process measures:

Working Alliance Inventory Short Form Revised (WAI-SR: Horvath, A. O. (1981) & Tracey, T. J., & Kokotovic, A. M. (1989). The WAI-SR is a 12-item scale measuring three domains of the therapeutic alliance. The WAI-SR is a patient-rated questionnaire. Patients rate items on a 5-point

Likert scale.

Demographic baseline measures: Age, gender, socio-economic status measured by educational level and income, years of being an informal caretaker.

Statistical Measures: 1) We will compare the outcome variables (DASS, PSS, SCS-12, MCS, ERQ, BRS, FFMQ-15, and WHO-5) correcting for multiple comparisons where appropriate using the Student t or Wilcoxon tests. Analysis of covariance (ANCOVA) will be conducted to assess whether CCT is related to changes between baseline and after 8 weeks of intervention in relation to psychological distress. We will also use a random-effects repeated measures analysis to examine the impact of the CCT intervention on psychological distress, adjusting for confounding variables (age, gender, socio-economic status, and years as informal caretaker). The repeated measure analysis approach accounts for the same individual's different outcome measures across different points in time without assuming either linear or curvilinear growth pattern. Cronbach alpha's will be computed to determine the internal consistency of our outcome measures.

The four measurement points allow to test whether changes in the proposed mediators are associated with changes in the proposed outcomes. This is a crucial condition in order to investigate mediators and possible mechanisms (Kazdin A.E., 2007). We will use structural equation modelling to examine the proposed mechanisms of CCT by testing the following action theories and conceptual theories simultaneously (Chen, H-T., 1994; Goldsmith et al., 2018). The current project assumes two conceptual theories, which will be tested: 1) changes in compassion for self and others will affect psychological distress (DASS) and 2) changes in emotion regulation skills of reappraisal and suppression (ERQ) will affect psychological distress (DASS). The action theories, that CCT changes 1) compassion for self (SCS-12) and others (MCS) and 2) emotion regulation skills of reappraisal and suppression (ERQ), will be tested. The indirect, direct and total effects will be estimated with 95% CI inspired by a framework suggested by Goldsmith et al., (2018). The statistical package M-Plus will be applied.

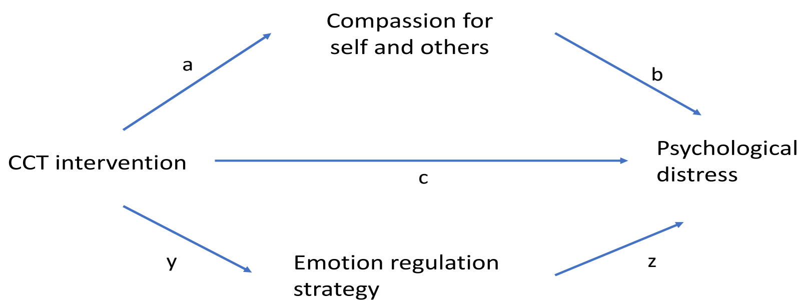

Plan for dissemination: Three research articles: 1) Mental Health interventions for caregivers of people with mental illness: A systematic review and meta-analysis, 2) CCT for Caregivers: A randomized controlled trial' and 3) 'Compassion for Caregivers: Can compassion be utilized as an emotion regulation strategy in decreasing psychological distress?'

Perspectives: It is paramount that preventive interventions for caregivers of mentally ill people are studied for their effectiveness and implemented, as it is widely known that these informal caregivers have a much greater risk of developing depression, anxiety, stress, and poor physical health. If the caregivers are not able to continue to care for their loved one, the detriment is not only to the caregiver, but also to the person being cared for. This proposal proposes a novel line of studying the effectiveness of a compassion training program as a preventive intervention program that may be of great benefit to informal caregivers, people with mental illness and the economic healthcare costs in Denmark.

References

Baer, R. A., Smith, G. T., Hopkins, J., Krietemeyer, J., & Toney, L. (2006). Using Self-Report Assessment Methods to Explore Facets of Mindfulness. *Assessment*, *13*(1), 27–45.

Bech P. *Clinical Psychometrics*, Wiley-Blackwell, Oxford, 2012.

Bedre Psykiatri – Landsforeningen for pårørende. Retrieved on February 2nd 2016.

http://www.bedrepsykiatri.dk

Borkovec, T.D. & Nau, S.D. (1972). Credibility of analogue therapy rationales. *Journal of Behavior Therapy and Experimental Psychiatry, 3, (4),* pp. 257-260.

Brito, G.P. (2014). *Cultivating healthy minds and open hearts. A mixed method controlled study on the psychological effects of compassion cultivation training in Chile*. A dissertation submitted in partial fulfillment of the requirements for the degree of Doctor of Philosophy in transpersonal psychology, Sofia University, Palo Alto, California, USA.

Brito-Pons, G., Campos, D., & Cebolla, A. (2018). Implicit or Explicit Compassion? Effects of Compassion Cultivation Training and Comparison with Mindfulness-based Stress Reduction. *Mindfulness*, 9, (5), pp. 1494-1508.

Center for Research on Health Care (CRHC) Data Center (2017). Retrieved on August 23rd, 2017 at http://www.pathwaysstudy.pitt.edu/codebook/bsi-sb.html.

Chapin H.L., Darnall, B.D., Seppala, E.M., Doty, J.R., Hah, J.M., & Mackey, S.C. (2014). Pilot study of a compassion meditation intervention in chronic pain. *Journal of Compassionate Care, 1*, (4), pp. 1-12.

Clark, M.C., Nicholas, J.M., Wassira, L.N., & Gutierrez, A.P. (2011). Psychological and biological indicators of depression in the caregiving population. *Biological Research for Nursing*.

Cohen, S., Kamarck, T., & Mermelstein, R (1983). A Global Measure of Perceived Stress. *Journal of*

Health and Social Behavior, Vol. 24, (4) pp. 385-396

Cosley, B.J., McKoy, S.K., Saslow, L.R., & Epel, E.S. (2010). Is compassion for others stress buffering? Consequences of compassion and social support for physiological reactivity to stress. *Journal of Experimental Social Psychology*, *46*, pp. 816-823.

Derogatis, L.R., Morrow, G.R., Fetting, J., Penman, D., Piasetsky, S., Schmale, A.M., Henrichs, M., & Carnicke, C.L.M. (1983). The prevalence of psychotic disorders among cancer patients. *JAMA*, *249,* (6), pp. 751-757.

Duggan, C., Parry, G., McMurran, M., Davidson, K., & Dennis, J. (2014). The recording of adverse events from psychological treatments in clinical trials: Evidence from a review of NIHR funded trials. *PMC, Trials*, *15,* (335), pp. 1-9.

Galante, J., Galante, I., Bekkers, M-J., & Gallacher, J. (2014). Effects of kindness-based meditation on health and well-being: A systematic review and meta-analysis. *Journal of Consulting and Clinical Psychology, 82,* (6), pp. 1101-1114.

Goetz, J.L. Keltner, D., Simon-Thomas, E (2010). Compassion: An evolutionary analysis and empirical review. *Psychological Bulletin*, 136, (3), pp. 351-374.

Goldin, P.R. & Jazaieri, H. (2017). The Oxford Handbook of Compassion Science Chapter 18: The Compassion Cultivation Training (CCT) Program. In Seppälä, E.M., Simon-Thomas, E., Brown, S.L., Worline, M.C., Cameron, D.C., & Doty, J.R (Eds.). *The Oxford Handbook of Compassion Science*.

Gross, J.J., & John, O.P. (2003). Individual differences in two emotion regulation processes: Implications for affect, relationships, and well-being. *Journal of personality and social psychology, 85,* (2), pp. 348-362.

Gu, J., Cavanaugh, K., Baer, R., & Strauss, C. (2017). An empirical examination of the factor structure of compassion. *PLOS ONE*, 12(2): e0172471. doi:10.1371/journal.pone.0172471.

Hoffman, S.G., Grossman, P., & Hinton, D.E. (2011). Loving-kindness and compassion meditation: Potential for psychological interventions. *Clinical Psychology Review, 31,* (7), pp. 1126-1132.

Horvath, A. O. (1981). *An exploratory study of the working alliance: Its measurement and relationship to therapy outcome*. Unpublished doctoral dissertation, University of British Columbia, Canada.

Hutcherson, C.A., Seppala, E.M., & Gross, J.J. (2008). Loving-kindness meditation increases social connectedness. *Emotion, 8,* (5), pp. 720-724.

Isenberg, N. (2015). *Compassion cultivation training (CCT) for family caregivers of persons with dementia, a qualitative pilot study*. Presented at the 2015 Annual Conference for Academy of Integrated Health and Medicine.

Jacobsen, R.H. (2011). *Analyse Rapport. Effekt af pårørendeinddragelse i behandlingen af mennesker med psykisk sygdom: Cost-benefit beregning*. Center for Economics and Business Research, Copenhagen Business School.

Jazaieri, H., Goldin, P. R., Simon-Thomas, E., Keltner, D., & Mendoza-Denton, R. (in prep). Predicting Compassionate Behavior: Application, development, and psychometric properties of the Multidimensional Compassion Scale.

Jazaieri, H., Jinpa, T., McGonigal, K., Rosenberg, E., Finkelstein, J., Simon-Thomas, E., & Goldin, P.R. (2013). Enhancing compassion: randomized controlled trial of a compassion cultivation training program. *Journal of Happiness Studies, 14,* pp. 1113-1126.

Jazaieri, H., McGonigal, K., Jinpa, T., Doty, J.R., Gross, J.J., & Goldin, P.R. (2014). A randomized controlled trial of compassion cultivation training: Effects on mindfulness, affect, and emotion regulation. *Motivation and Emotion, 38,* pp. 23-35

Jazaieri, H., Lee, I.A., McGonigal, K., Jinpa, T., Doty, J.R., Gross, J.J., & Goldin, P.R., (2015). A wandering mind is a less caring mind: Daily experience sampling during compassion meditation training. *The Journal of Positive Psychology*, Routledge.

Jazaieri, H., McGonigal, K., Lee, I. A., Jinpa, T., Doty, J. R., Gross, J. J., & Goldin, P. R. (2017). Altering the trajectory of affect and affect regulation: The impact of compassion training. *Mindfulness*.

Advance online publication. doi: 10.1007/s12671-017-0773-3

Jinpa, T. (2010*). Compassion cultivation training program (CCT program): An eight-week course on cultivation a compassionate heart and mindset. Instructor's Manual*. The Center for Compassion and Altruism Research and Education (CCARE). Stanford Institute for Neuro-Innovation and Translational Neuroscience, Stanford University (Unpublished).

Kirby, J.N. (2016). Compassion interventions: The programmes, the evidence, and implications for research and practice. *Psychology and Psychotherapy, Research and Practice*. DOI:10.1111/papt.12104.

Knight, B.G., Lutzky, S.M., & Macofsky-Urban, F. (1993). A meta-analytic review of interventions for caregiver distress: Recommendations for future research. *The Gerontologist, 33,* (2), pp. 240-248.

Kok, B.E., & Singer, T. (2016). Effects on contemplative dyads on engagements and perceived social connectedness over 9 months of mental training: A randomized clinical trial. *JAMA Psychiatry*

Kuhlemann, S.M., Bürger, A., Esser, G., & Hammerle, F. (2015). A mindfulness-based stress prevention training for medical students (MediMind): Study protocol for a randomized controlled trial. *Trials, 16,* (40), pp. 1-11.

Lovibond, S.H. & Lovibond, P.F. (1995). *Manual for the Depression Anxiety Stress Scales. (2nd. Ed.)* Sydney: Psychology Foundation.

Lykins, E.R.B., & Baer, R.A. (2009). Psychological functioning in a sample of long-term practitioners of mindfulness meditation. *Journal of Cognitive Psychotherapy, 23,* (3), pp. 226-241.

Lwi, S.J., Ford, B.Q., Casey, J.J., Miller, B.L., & Levenson, R.W. (2017). Poor caregiver mental health predicts mortality of patients with neurodegenerative disease. *PNAS, 114,* (28), pp. 7319-7324.

Northouse, L.L., Katapodi, M.C., Song, L., Zhang, L., & Mood, D.W. (2010). Interventions with Family Caregivers of Cancer Patients: Meta-Analysis of Randomized Trials. *CA Cancer J Clin, 60*, pp. 317-339.

Olesen, J., Gustavsson, A., Svensson, M., Wittchen, H.U., & Jönnson, B. (2012). The economic cost of brain disorders in Europe. *European Journal of Neurology, 19,* pp. 155- 162.

Psykiatrifonden (The Mental Health Fund), 2017. *Psykisk sundhed i Danmark: Samfundsomkostninger.* Retrieved on August 23rd, 2017 at http://www.psykiatrifonden.dk/viden/fakta.aspx.

Raes, F., Pommier, E., Neff, K. D., & Van Gucht, D. (2011). Construction and factorial validation of a short form of the Self-Compassion Scale. *Clinical Psychology & Psychotherapy*. 18, 250-255.

Scarlet, J., Altmeyer, N., Knier, S., & Harpin, E. (2017). The effects of compassion cultivation training (CCT) on health-care workers. *Clinical Psychologist, 21,* pp. 116-124.

Shonin, E., Van Gordon, W., Compare, A., Zangeneh, M., & Griffiths, M.D. (2015). Buddhist-derived loving-kindness and compassion meditation for the treatment of psychopathology: A systematic reveiw. *Mindfulness*, 6, pp. 1161-1180.

Singer, T. & Klimicki, O.M. (2014). Empathy and Compassion. *Current Biology*, 24, (18), pp. 1-4.

Smith, B. W., Dalen, J., Wiggins, K., Tooley, E., Christopher, P., Bernard, J. (2008). The Brief Resilience Scale: Assessing the ability to bounce back. *International Journal of Behavioral Medicine*, 15, (3), pp. 194-200.

Sorrell, J.M. (2014). Moving beyond caregiver burden. *Journal of Psychosocial Nursing, 52,* (3), 15-18.

Sörensen, S., Pinquart, M, & Duberstein, P. (2002). How effective are interventions with caregivers? An updated meta-analysis. *The Gerontologist, 42,* (3), pp. 356-372.

Stansfeld, S., Smuk, M., Onwumere, J., Clark, C., Pike, C., McManus, S., Harris, J., & Bebbington, P. (2014). Stressors and common mental disorders in informal carer – An analysis of the English adult psychiatric morbidity survey 2007. *Social Science and Medicine, 120,* pp. 190-198.

The Lancet Editorial (2017). Who cares for the carer? *The Lancet, 389,* p. 763.

Tracey, T. J. and Kokotovic, A. M. 1989. Factor structure of the Working Alliance Inventory. *Psychological Assessment*, 1: 207–210.

Yesufu-Udechuku, A., Harrison, B., Mayo-Wilson, E., Young, N., Woodhams, P., Shiers, D., Kuipers, & E., Kendall, T. (2015). Interventions to improve the experience of caring for people with severe mental illness: Systematic review and meta-analysis. *The British Journal of Psychiatry, 206,* pp. 268-274.

Wright, V., & Pendry, B. (2016). Compassion and its role in the clinical encounter – An argument for compassion training. *Journal of Herbal Medicine*, *149,* pp. 1-6.

Statistical Analysis Plan: SAP_JAMA

<u>Statistical Measures:</u> 1) We will compare the outcome variables (DASS, PSS, SCS-12, MCS, ERQ, BRS, FFMQ-15, and WHO-5) correcting for multiple comparisons where appropriate using the Student t or Wilcoxon tests. Analysis of covariance (ANCOVA) will be conducted to assess whether CCT is related to changes between baseline and after 8 weeks of intervention in relation to psychological distress. We will also use a random-effects repeated measures analysis to examine the impact of the CCT intervention on psychological distress, adjusting for confounding variables (age, gender, socio-economic status, and years as informal caretaker). The repeated measure analysis approach accounts for the same individual's different outcome measures across different points in time without assuming either linear or curvilinear growth pattern. Cronbach alpha's will be computed to determine the internal consistency of our outcome measures.

The four measurement points allow to test whether changes in the proposed mediators are associated with changes in the proposed outcomes. This is a crucial condition in order to investigate mediators and possible mechanisms (Kazdin A.E., 2007). We will use structural equation modelling to examine the proposed mechanisms of CCT by testing the following action theories and conceptual theories simultaneously (Chen, H-T., 1994; Goldsmith et al., 2018). The current project assumes two conceptual theories, which will be tested: 1) changes in compassion for self and others will affect psychological distress (DASS) and 2) changes in emotion regulation skills of reappraisal and suppression (ERQ) will affect psychological distress (DASS). The action theories, that CCT changes 1) compassion for self (SCS-12) and others (MCS) and 2) emotion regulation skills of reappraisal and suppression (ERQ), will be tested. The indirect, direct and total effects will be estimated with 95% CI inspired by a framework suggested by Goldsmith et al., (2018). The statistical package M-Plus will be applied.

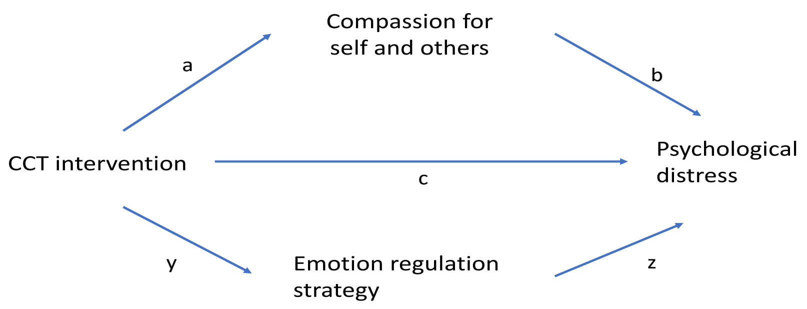
Compassion for self and others
a
b
CCT intervention
c
Psychological distress
y
Emotion regulation strategy
z
