# Supplementary material for: Effect of a Compassion Cultivation Training Program for Caregivers of People With Mental Illness in Denmark: A Randomized Clinical Trial
Source: JAMA Netw Open. 2021 Mar 8;4(3):e211020. doi: 10.1001/jamanetworkopen.2021.1020 (PMC7941195; doi:10.1001/jamanetworkopen.2021.1020)
Supplement: Supplement 2. — eTable 1. Steps of the Compassion Cultivation Training Program eTable 2. Loss to 6-Month Follow-up Analysis of Included and Missing Participants in Both the CCT Intervention and Waitlist Control Groups at 6-Month Follow-up eTable 3. Loss to 6-Month Follow-up Analysis of Included and Missing Participants in the CCT Intervention Group at 6-Month Follow-up eTable 4. Loss to 6-Month Follow-up Analysis of Included and Missing Participants in Waitlist Control Group at 6-Month Follow-up eTable 5. Sensitivity Analysis of Primary Outcome of Psychological Distress eTable 6. Sensitivity Analysis of Secondary Outcome of Overall Well-Being [file jamanetwopen-e211020-s002.pdf]

Supplemental Online Content

Hansen NH, Juul L, Pallesen KJ, Fjorback LO. Effect of a compassion cultivation training program for caregivers of people with mental illness in Denmark: a randomized clinical trial. *JAMA Netw Open*. 2021;4(3):e211020. doi:10.1001/jamanetworkopen.2021.1020

eTable 1. Steps of the Compassion Cultivation Training Program
eTable 2. Loss to 6-Month Follow-up Analysis of Included and Missing Participants in Both the CCT Intervention and Waitlist Control Groups at 6-Month Follow-up
eTable 3. Loss to 6-Month Follow-up Analysis of Included and Missing Participants in the CCT Intervention Group at 6-Month Follow-up
eTable 4. Loss to 6-Month Follow-up Analysis of Included and Missing Participants in Waitlist Control Group at 6-Month Follow-up
eTable 5. Sensitivity Analysis of Primary Outcome of Psychological Distress
eTable 6. Sensitivity Analysis of Secondary Outcome of Overall Well-Being

This supplemental material has been provided by the authors to give readers additional information about their work.

© 2021 Hansen NH et al. *JAMA Network Open.*

eTable 1. Steps of the Compassion Cultivation Training Program

**Week 1**	Introductions and learning to focus and settle the mind
**Week 2**	Cultivating loving kindness and compassion for a loved one
**Week 3**	Training compassion for one's own suffering
**Week 4**	Training loving-kindness for one's own suffering
**Week 5**	Cultivating compassion towards others through embracing shared common humanity and appreciating the interconnectedness of all human beings
**Week 6**	Training compassion towards all sentient beings
**Week 7**	Training active compassion using a Tonglen practice (it means to give and take) where participants imagine taking others' suffering away, and offering them all that is good in oneself
**Week 8**	Closure and training an integrated compassion practice

© 2021 Hansen NH et al. *JAMA Network Open.*

eTable 2. Loss to 6-Month Follow-up Analysis of Included and Missing Participants in Both the CCT Intervention and Waitlist Control Groups at 6-Month Follow-up

	**Included**	**Missing**	**P-value**
**Sex, n (%)**	n = 116	n = 34	
Female	111 (88.10)	30 (88.24)	0.982
Male	15 (11.90)	4 (11.76)	
**Age, mean (SD)**	51.90 (11.65)	55.34 (15.13)	0.155
**Educational level, n (%)**			
No highschool	1 (0.79)	1 (2.86)	
Highschool	5 (3.97)	1 (2.86)	
Trade school	12 (9.52)	3 (8.57)	
Short continuing education	8 (6.35)	3 (8.57)	
Medium continuing education	54 (42.86)	14 (40.00)	
Long continuing education	44 (34.92)	11 (31.43)	
Ph.D.	2 (1.59)	1 (2.86)	
Other	0 (0.00)	1 (2.86)	0.630
**Years of Caretaking**			
0-5	32 (25.81)	12 (34.29)	
5-10	38 (30.65)	5 (14.29)	
10-15	17 (13.71)	4 (11.43)	
15-20	10 (8.06)	4 (11.43)	
> 20	27 (21.77)	10 (28.57)	0.353
**DASS Depression, mean (SD)**	10.90 (8.44)	10.64 (8.81)	0.874
**DASS Anxiety, mean (SD)**	6.90 (6.08)	6.35 (5.19)	0.632
**DASS Stress, mean (SD)**	15.79 (7.66)	13.88 (7.50)	0.200
**PSS, mean (SD)**	22.19 (6.45)	21.18 (6.07)	0.385
**WHO-5, mean (SD)**	44.84 (17.47)	45.94 (17.47)	0.776
**BRS, mean (SD)**	3.00 (.83)	3.14 (.68)	0.375
**ERQ Reappraisal, mean (SD)**	25.06 (7.00)	25.00 (6.96)	0.963
**ERQ Emotion Suppression, mean (SD)**	12.42 (4.67)	12.29 (5.18)	0.895

*Test of no difference between participants answering at 6 months follow-up and participants lost to follow-up. Pearson's χ^2 categorical variables, while t-test was used for continuing variables.

** Depression Anxiety Stress Scale (DASS), Perceived Stress Scale (PSS), World Health Organization (5) Well Being Index (WHO-5), Brief Resilience Scale (BRS), Emotion Regulation Questionnaire (ERQ)

© 2021 Hansen NH et al. *JAMA Network Open.*

eTable 3. Loss to 6-Month Follow-up Analysis of Included and Missing Participants in the CCT Intervention Group at 6-Month Follow-up

	CCT		
	**Included**	**Missing**	**P-value**
**Sex, n (%)**			
Female	46 (85.19)	21 (87.50)	
Male	8 (14.81)	3 (12.50)	0.79
**Age, mean (SD)**	54.98 (11.62)	57.97 (16.68)	0.37
**Educational level, n (%)**			
No highschool	0 (0.00)	1 (4.00)	
Highschool	3 (5.56)	1 (4.00)	
Trade school	3 (5.56)	2 (8.00)	
Short continuing education	6 (11.11)	2 (8.00)	
Medium continuing education	31 (57.41)	12 (48.00)	
Long continuing education	11 (20.37)	6 (24.00)	
Ph.D.	0 (0.00)	0 (0.00)	
Other	0 (0.00)	1 (4.00)	0.53
**Years of Caretaking**			
0-5	13 (24.53)	9 (36.00)	
5-10	20 (37.74)	3 (12.00)	
10-15	3 (5.66)	2 (8.00)	
15-20	5 (9.43)	4 (16.00)	
> 20	12 (22.64)	7 (28.00)	0.23
**DASS Depression, mean (SD)**	10.46 (8.59)	11.83 (8.90)	0.52
**DASS Anxiety, mean (SD)**	7.12 (6.82)	6.42 (5.83)	0.67
**DASS Stress, mean (SD)**	15.50 (7.96)	13.84 (7.80)	0.39
**PSS, mean (SD)**	20.96 (6.37)	21.72 (5.44)	0.61
**WHO-5, mean (SD)**	47.00 (22.82)	46.40 (16.41)	0.91
**BRS, mean (SD)**	3.11 (.81)	3.13 (.73)	0.90
**ERQ Reappraisal, mean (SD)**	25.87 (7.04)	24.92 (7.75)	0.59
**ERQ Emotion Suppression, mean (SD)**	12.63 (4.50)	12.80 (5.65)	0.75

*Test of no difference between participants answering at 6 months follow-up and participants lost to follow-up. Pearson's χ^2 categorical variables, while t-test was used for continuing variables.
** Depression Anxiety Stress Scale (DASS), Perceived Stress Scale (PSS), World Health Organization (5) Well Being Index (WHO-5), Brief Resilience Scale (BRS), Emotion Regulation Questionnaire (ERQ)

© 2021 Hansen NH et al. *JAMA Network Open.*

eTable 4. Loss to 6-Month Follow-up Analysis of Included and Missing Participants in Waitlist Control Group at 6-Month Follow-up

	Control		
	**Included**	**Missing**	**P-value**
**Sex, n (%)**			
Female	65 (90.28)	9 (90.00)	
Male	7 (9.72)	1 (10.00)	0.98
**Age, mean (SD)**	49.59 (11.21)	49.04 (8.13)	0.88
**Educational level, n (%)**			
No highschool	1 (1.39)	0 (0.00)	
Highschool	2 (2.78)	1 (10.00)	
Trade school	9 (12.50)	1 (10.00)	
Short continuing education	2 (2.78)	2 (20.00)	
Medium continuing education	23 (31.94)	14 (40.00)	
Long continuing education	33 (45.83)	5 (50.00)	
Ph.D.	2 (2.78)	1 (10.00)	
Other	0 (0.00)	0 (0.00)	0.76
**Years of Caretaking**			
0-5	19 (26.76)	3 (30.00)	
5-10	18 (25.35)	2 (20.00)	
10-15	14 (19.72)	2 (20.00)	
15-20	5 (7.04)	0 (0.00)	
> 20	15 (21.13)	3 (30.00)	0.89
**DASS Depression, mean (SD)**	11.23 (8.37)	7.44 (8.16)	0.20
**DASS Anxiety, mean (SD)**	6.75 (5.55)	6.2 (3.43)	0.76
**DASS Stress, mean (SD)**	16.00 (7.47)	14.00 (7.04)	0.45
**PSS, mean (SD)**	23.11 (6.40)	19.44 (7.70)	0.12
**WHO-5, mean (SD)**	43.27 (19.09)	44.80 (20.81)	0.81
**BRS, mean (SD)**	2.92 (.83)	3.15 (.60)	0.40
**ERQ Reappraisal, mean (SD)**	24.46 (6.96)	25.22 (4.41)	0.75
**ERQ Emotion Suppression, mean (SD)**	12.25 (4.44)	10.89 (3.44)	0.38

*Test of no difference between participants answering at 6 months follow-up and participants lost to follow-up. Pearson's χ^2 categorical variables, while t-test was used for continuing variables.

** Depression Anxiety Stress Scale (DASS), Perceived Stress Scale (PSS), World Health Organization (5) Well Being Index (WHO-5), Brief Resilience Scale (BRS), Emotion Regulation Questionnaire (ERQ)

© 2021 Hansen NH et al. *JAMA Network Open.*

eTable 5. Sensitivity Analysis of Primary Outcome of Psychological Distress

	CCT			Control		
	Change score	CI 95%	p-value	Change score	CI 95%	p-value
			**+ 0.2 SD**			
DASS Depression						
Post	-3.52	-5.94 - -1.10	0.004	-3.78	-6.22 - -1.35	0.002
3 months	-4-04	-6.46 - -1.62	0.001	-4.43	-6.86 - -1.99	0.000
6 months	-4.26	-6.68 - -1.83	0.001	-4.95	-7.39 - -2.52	0.000
DASS Anxiety						
Post	-2.02	-3.66 - -.39	0.015	-2.26	-3.90 - -.63	0.007
3 months	-2.47	-4.11 - -.84	0.003	-2.75	-4.38 - -1.12	0.001
6 months	-2.08	-3.72 - -.45	0.012	-2.57	-4.21 - -.94	0.002
DASS Stress						
Post	-4.19	-6.59 - -1.79	0.001	-4.46	-6.87 - -2.06	0.000
3 months	-4.08	-6.48 - -1.69	0.001	-4.42	-6.83 - -2.02	0.000
6 months	-3.94	-6.34 - -1.55	0.001	- 4.60	-7.00 - -2.20	0.000
			-**0.2 SD**			
DASS Depression						
Post	-3.91	-6.34 - -1.47	0.002	-3.64	-6.06 - -1.22	0.003
3 months	-4.67	-7.11 - -2.24	0.000	-4.29	-6.71 - -1.87	0.001
6 months	-5.32	-7.76 - -2.89	0.000	-4.63	-7.05 - -2.21	0.000
DASS Anxiety						
Post	-2.22	-3.85 - -.59	0.008	-1.98	-3.61 - -.35	0.017
3 months	-2.84	-4.47 - -1.21	0.001	-2.56	-4.19 - -.93	0.002
6 months	-2.75	-4.38 - -1.12	0.001	-2.26	-3.89 - -.63	0.007
DASS Stress						
Post	-4.69	-7.11 - -2.28	0.000	-4.42	-6.82 - -2.02	0.000
3 months	-4.68	-7.09 - -2.26	0.000	-4.34	-6.74 - -1.93	0.000
6 months	-4.95	-7.37 - -2.54	0.000	-4.29	-6.70 - -1.89	0.000

* Missing outcomes were substituted with the model-based prediction adding or subtracting 0.2 SD in the intervention or control arm

** Depression Anxiety Stress Scale (DASS)

© 2021 Hansen NH et al. *JAMA Network Open.*

eTable 6. Sensitivity Analysis of Secondary Outcome of Overall Well-Being

	CCT			Control		
	Change score	CI 95%	p-value	Change score	CI 95%	p-value
			**+ 0.2 SD**			
WHO-5						
Post	10.81	5.04 – 16.57	0.000	10.16	4.42 – 15.91	0.001
3 months	8.95	3.18 – 14.71	0.002	7.98	2.24 – 13.72	0.006
6 months	9.80	4.03 – 15.56	0.001	8.14	2.39 – 13.98	0.005
BRS						
Post	.27	.08 - .46	0.005	.25	.06 - .43	0.009
3 months	.36	.17 - .55	0.000	.32	.14 - .51	0.001
6 months	.41	.22 - .59	0.000	.34	.16 - .53	0.000
PSS						
Post	-3.78	-5.51 - -1.91	0.000	-4.00	-5.81 - -2.19	0.000
3 months	-2.87	-4.67 - -1.07	0.002	-3.23	-5.04 - -1.42	0.000
6 months	-1.96	-3.76 - -.16	0.033	-2.54	-4.36 - - .73	0.006
ERQ-Reappraisal						
Post	4.44	2.61 – 6.27	0.000	4.15	2.34 – 5.97	0.000
3 months	3.31	1.48 – 5.14	0.000	2.96	1.15 – 4.76	0.001
6 months	2.80	.97 – 4.63	0.003	2.22	.41 – 4.03	0.016
ERQ-Suppression						
Post	-1.33	-2.65 - -.01	0.049	-1.48	-2.81 - -.16	0.028
3 months	-1.18	-2.50 - .14	0.081	-1.40	-2.72 - -.07	0.038
6 months	-1.41	-2.73 - -.09	0.036	-1.80	-3.11 - -.47	0.008
			**-0.2 SD**			
WHO-5						
Post	9.83	4.10 – 15.56	0.001	-3.64	-6.06 - -1.22	0.003
3 months	7.43	1.70 – 13.15	0.011	-4.29	-6.71 - -1.87	0.001
6 months	7.30	1.57 – 13.03	0.002	-4.63	-7.05 - -2.21	0.000
BRS						
Post	.24	.05 - .43	0.011	.26	.08 - .45	0.006
3 months	.30	.12 - .49	0.001	.34	.15 - .53	0.000
6 months	.21	.13 - .50	0.001	.38	.19 - .56	0.000
PSS						
Post	-4.08	-5.89 - -2.26	0.000	-3.79	-5.59 - - 1.98	0.000
3 months	-3.42	-5.24 - -1.61	0.000	-3.06	-4.96 - - 1.26	0.001
6 months	-2.85	-4.66 - -1.03	0.002	-2.26	-4.06 - -.46	0.014
ERQ-Reappraisal						
Post	4.07	2.26 – 5.89	0.000	4.36	2.54 – 6.19	0.000

© 2021 Hansen NH et al. *JAMA Network Open.*

3 months	2.76	.94 – 4.57	0.003	3.10	1.27 – 4.93	0.001
6 months	1.92	.10 – 3.73	0.038	2.50	.67 – 4.33	0.007
ERQ-Suppression						
Post	-1.60	-2.92 - -.28	0.018	-1.44	-2.75 - -.12	0.032
3 months	-1.55	-2.87 - -.23	0.022	-1.33	-2.64 - -.01	0.049
6 months	-2.00	-3.32 - -.68	0.003	-1.63	-2.94 - -.31	0.015

* Missing outcomes were substituted with the model-based prediction adding or subtracting 0.2 SD in the intervention or control arm

** World Health Organization (5) Well Being Index (WHO-5), Brief Resilience Scale (BRS), Perceived Stress Scale (PSS), Emotion Regulation Questionnaire (ERQ)

© 2021 Hansen NH et al. *JAMA Network Open.*
