# Supplementary material for: Effect of a Compassion Cultivation Training Program for Caregivers of People With Mental Illness in Denmark: A Randomized Clinical Trial
Source: JAMA Netw Open. 2021 Mar 8;4(3):e211020. doi: 10.1001/jamanetworkopen.2021.1020 (PMC7941195; doi:10.1001/jamanetworkopen.2021.1020)
Supplement: Supplement 3. — Data Sharing Statement [file jamanetwopen-e211020-s003.pdf]

Data Sharing Statement

Hansen. Effect of a Compassion Cultivation Training Program for Caregivers of People With Mental Illness in Denmark. *JAMA Netw Open*. Published March 08, 2021. doi:10.1001/jamanetworkopen.2021.1020

Data
Data available: No
